# Knowledge, attitudes, and practices regarding advance care planning among emergency department healthcare providers in China: a multicenter cross-sectional study

**DOI:** 10.3389/fpubh.2026.1815766

**Published:** 2026-06-15

**Authors:** Wenya Bi, Xiufang Zhang, Chengwen Hu

**Affiliations:** School of Nursing, Anhui Medical University, Hefei, Anhui, China

**Keywords:** advance care planning, cross-sectional survey, emergency department, healthcare providers, knowledge, attitudes, practices

## Abstract

**Background:**

Emergency departments frequently treat critically ill and end-of-life patients; therefore, healthcare providers’ knowledge of advance care planning (ACP) and their communication skills are crucial for identifying patients’ care preferences. This study aims to investigate the current status of ACP knowledge, attitudes, and practice intentions among healthcare providers in Chinese emergency departments, together with the factors influencing them.

**Methods:**

This study conducted a multicenter, cross-sectional online survey in December 2024 among physicians and nurses in the emergency departments of 54 secondary and tertiary public general hospitals in Anhui Province, China. Descriptive statistics were used to analyze the levels of knowledge, attitudes, and practice intentions regarding ACP; Pearson correlation analysis was used to explore the relationships among these three factors; and independent-samples t-tests, one-way ANOVA, and multiple linear regression analysis were used to identify relevant influencing factors.

**Results:**

Of 720 submitted questionnaires, 686 valid responses were included in the final analysis. The mean scores for ACP knowledge, attitudes, and practice intentions were 7.25 ± 5.37, 73.91 ± 25.24, and 21.56 ± 10.31, respectively. Multiple linear regression analysis indicated that gender, age, years of experience in the emergency department, educational attainment, exposure to ACP-related information, palliative/hospice care education, and experience with end-of-life care correlated with ACP knowledge; age, years of experience in the emergency department, occupation, exposure to ACP-related information, and experience with end-of-life care correlated with ACP attitudes; and age, hospital grade, and exposure to ACP-related information correlated with ACP practice intentions.

**Conclusion:**

Emergency department healthcare providers recognized the value of ACP, but their ACP-related knowledge and practice intentions remained limited. Emergency-specific training, standardized communication and referral procedures, and clearer legal and ethical safeguards are needed to support feasible ACP-related practice in emergency settings.

## 1 Introduction

Advance care planning (ACP) is the process through which persons, regardless of age or health status, articulate and deliberate their future healthcare preferences with family members and healthcare professionals prior to any cognitive or communicative decline (1, 2). Research data (3, 4) suggests that 75.0% of patients aged 65 and over visited the emergency room within the 6 months prior to their death. Furthermore, 83.9% of cancer patients consistently pursued emergency care during the terminal period to alleviate symptoms like pain and dyspnea. The early incorporation of palliative care-related interventions in emergency settings may mitigate physical and emotional distress for patients and families, improve quality of life and care satisfaction, optimize healthcare resource utilization, shorten hospital admissions, reduce costs, and alleviate financial strain on families (5, 6). ACP is closely related to goals-of-care communication and the documentation of future care preferences. Therefore, understanding the potential role of ACP-related work in emergency settings is important. Healthcare providers, as direct caregivers to terminally ill patients, must honor patients’ autonomy in decision-making and support their desire to die with dignity (7). They should be prepared to recognize ACP-related needs, communicate appropriately when feasible, and facilitate referral to palliative care or ethics consultation services. However, the emergency department environment is characterized by urgency, high patient turnover, and rapidly changing conditions (8). Healthcare providers frequently face the challenge of making critical decisions for critically ill or terminally ill patients within limited time frames. It is important to recognize that ACP is typically more suitable in circumstances where a patient’s condition is stable and there is adequate time for communication and decision-making; this does not suggest that a comprehensive ACP discussion should be commenced immediately in all emergency resuscitation or acute crisis scenarios. Emergency departments often encounter older adult patients, those with advanced cancer, individuals suffering from chronic end-stage diseases, or frequent visitors; these patients may possess unrecognized future care preferences and require assistance with end-of-life decision-making. Consequently, when emergency department personnel are equipped with knowledge of ACP and fundamental communication skills, they can more effectively identify patients appropriate for further ACP evaluation, assist in documenting patient preferences, and ensure smooth coordination with referrals to palliative care and ethics consultation services. In situations of clinical ambiguity or substantial ethical dilemmas, ethical consultation and interdisciplinary discourse may be preferable to the rapid commencement of ACP.

In China, the therapeutic application of ACP is still in its preliminary stage, with legal protections, ethical frameworks, standardized protocols, and public awareness inadequately developed. Simultaneously, medical decision-making among Chinese patients is frequently shaped by familial involvement, traditional concepts of filial piety, and cultural taboos regarding death, hindering patients from articulating their individual preferences in emergency situations. Healthcare providers in the emergency department encounter a clinical setting marked by increased intensity, heightened uncertainty, and elevated communication hazards. Therefore, evaluating healthcare providers’ knowledge, attitudes, and practices regarding ACP in the context of Chinese emergency departments not only helps assess their level of ACP readiness but also provides a basis for establishing ACP training, communication, and referral mechanisms tailored to the Chinese emergency setting.

Although research has examined ACP understanding and attitudes among personnel in community-based, oncology, and long-term care facilities (9–11), studies focused on the emergency department context are limited. Previous research (12) have concentrated on emergency department nurses; however, there is insufficient comprehension of the knowledge, attitudes, and practices of ACP among emergency physicians and the nursing workforce at large, along with the factors that influence these elements. Emergency physicians and nurses fulfill separate but complementary functions in patient evaluation, clinical decision-making, symptom treatment, family communication, and care coordination; thus, it is essential to incorporate both groups in the study.

Accordingly, this study surveyed physicians and nurses in the emergency departments of secondary and tertiary public general hospitals in Anhui Province, China, to evaluate their knowledge, attitudes, and practices concerning ACP and to analyze the influencing factors involved. The study seeks to provide evidence for ACP-related education and training, clinical communication processes, and the development of referral systems linking emergency departments with palliative care and ethics consultation services in China.

## 2 Methods

### 2.1 Study design

This research is a multicenter, cross-sectional online survey done in December 2024 among healthcare providers in the emergency departments of secondary and tertiary public general hospitals in Anhui Province, China. Since strict probability-based random sampling is difficult to implement in practical surveys, this study adopted a two-stage sampling strategy. At the hospital level, stratified quota sampling was used, with the 16 prefecture-level cities in Anhui Province serving as strata. Taking into account hospital grade, geographic coverage, the presence of emergency departments, feasibility of survey collaboration, and willingness to participate, 2–4 secondary or tertiary public general hospitals were selected from each prefecture-level city. At the participant level, convenience sampling was employed. Designated contacts from the emergency departments of each participating hospital distributed the survey link to emergency department physicians and nurses who met the inclusion criteria.

### 2.2 Participants

Eligible participants were physicians and nurses working in the emergency departments of the participating hospitals. The following are requirements for inclusion: (a) Having a current certificate of practice as a doctor or nurse; (b) Having at least a year’s experience in emergency medicine or nursing; (c) Voluntary involvement in the study. Exclusion criteria: (a) During the survey period, physicians and nurses who were participating in advanced training or standardized training rotations in the emergency department. (b) Medical personnel working in emergency rooms who are away from work because of leave, outside training, or other circumstances. The sample size estimate was based on the results of a pilot study conducted prior to the main survey. The pilot study included a total of 30 emergency department healthcare professionals, and the standard deviation of the total score on the ACP Knowledge, Attitudes, and Practices Scale was 18.28, with a specified error *δ* of roughly 1.42. Using the sample size calculation formula (13, 14) with $n={\left(u_{\alpha /2}\times \sigma /\delta \right)}^{2}$ for cross-sectional studies and accounting for the margin of error, the minimum required sample size was calculated to be approximately 637 participants.

### 2.3 Data collection

The survey instrument for this study consisted of two segments: a general information questionnaire and a questionnaire assessing healthcare professionals’ knowledge, attitudes, and practice intentions on ACP. The general information questionnaire encompassed gender, age, occupation, professional title, educational background, awareness of ACP-related information and palliative/hospice care education experience, and experience in managing death. Professional title in this study are classified according to the Chinese healthcare professional title system. Junior titles primarily include resident physicians and junior nurses; intermediate titles primarily include attending physicians and senior nurses; associate senior and higher titles include associate chief physicians, chief physicians, associate chief nurses, and chief nurses; palliative/hospice care education experience refers to the research subjects’ prior education or training related to end-of-life care, palliative care, hospice care, or end-of-life communication. Given that these concepts often overlap in practice within clinical curricula and continuing education programs in China, this study uses this variable to reflect the research subjects’ prior exposure to education related to end-of-life care. Variable selection was informed by a literature review (15, 16) and the study objectives, with the intent to investigate the potential impact of demographic variables, professional background, specialized training experience, and particular clinical experience on ACP knowledge, attitudes, and practices. The ACP knowledge, attitude, and practices questionnaire for healthcare professionals utilized the “Advance Care Planning Knowledge, Attitudes, and Practice Questionnaire for Oncology Healthcare Professionals” created by Huang et al. (17). The questionnaire attained a Cronbach’s alpha coefficient of 0.963 and consisted of three sections: knowledge, attitudes, and practices, encompassing a total of 48 items. The knowledge dimension consists of 15 items, awarding 1 point for each correct answer and 0 points for wrong or uncertain responses. The theoretical possible score range for the knowledge dimension is 0–15, with higher scores indicating higher ACP knowledge. The theoretical possible score range for the attitude dimension is 24–120, with higher scores indicating more positive attitudes toward ACP. The theoretical possible score range for the practice intention dimension is 9–45, with higher scores indicating stronger willingness to participate in ACP-related practices. The theoretical possible total score range is 33–180.

Originally intended for cancer departments, the questionnaire’s fundamental material is relevant across all departments and appropriate for all medical environments concerning end-of-life care. Emergency departments, similar to oncology departments, often face communication and care requirements for many terminally ill patients, exhibiting significant overlap in advance care planning basic principles and practice approach. Consequently, amending this questionnaire for the emergency setting is both rational and practicable. The revision process encompassed: (a) Content adaptation: Substituting all occurrences of “terminal cancer patients” in the original questionnaire with “emergency department patients” or “terminally ill patients”; (b) Expert review: Five specialists (three in emergency medicine and two in palliative care) assessed the content validity of the revised items, with specific phrasings refined according to their feedback; (c) Pilot survey: A pilot survey was administered to 30 healthcare providers in the emergency department to assess the questionnaire’s clarity and practicality. Subsequent to these modifications, the questionnaire attained a comprehensive Cronbach’s *α* coefficient of 0.963 in this study, with dimension coefficients as follows: Knowledge Dimension 0.937, Attitude Dimension 0.976, and Practice Intention Dimension 0.947. The full questionnaire is provided in Supplementary Appendix 1.

This research gathered data through an online survey disseminated via the QuestionStar platform. This study was conducted as a closed online survey. The survey link was not publicly posted on any public website or social media platform; instead, it was distributed by designated contacts in the emergency departments of each participating hospital to physicians and nurses who met the inclusion criteria. To minimize duplicate submissions, the platform was configured to allow only one submission per IP address. The research team did not conduct independent log file analysis outside the platform. During the data cleaning phase, surveys with abnormal completion times, repetitive responses, or logical inconsistencies were excluded. Prior to completing the questionnaire, participants were mandated to review the study objectives, the anonymity statement, the intended data usage, and the principle of voluntary participation; they were subsequently permitted to advance to the main questionnaire upon confirming their informed consent.

### 2.4 Data analysis

The data for this study were exported from the QuestionStar online survey platform to Microsoft Excel for first verification and subsequently entered into SPSS 25.0 for statistical analysis. Before conducting formal analysis, the researchers assessed the data for completeness, duration of completion, response patterns, and logical coherence. Questionnaires with completion durations under 180 s, responses displaying discernible trends, or logical discrepancies were omitted. The questionnaire was structured to require the completion of all items prior to submission; hence, there were no item-level missing values in the valid surveys. During the data cleaning phase, incomplete surveys were removed, and no imputation for missing values was conducted.

The statistical approaches employed comprised descriptive statistics, correlation analysis, independent samples t-tests, one-way analysis of variance (ANOVA), and multiple linear regression analysis. The significance level was established at *α* = 0.05, with *p* < 0.05 denoting statistically significant differences. (a) Descriptive statistical analysis: Categorical variables are described using frequencies and percentages, such as gender, age groups, occupation, hospital grade, educational level and professional title, among others. Continuous variables are described using means and standard deviations, such as scores for ACP knowledge, attitudes, and practice intentions. (b) Correlation analysis: Pearson correlation analysis was employed to examine the association among ACP knowledge, attitudes, and practice intentions. (c) Univariate Analysis: To compare ACP knowledge, attitudes, and practice intentions scores across different demographic and occupational characteristics, an independent samples *t*-test was used for two variables, and one-way analysis of variance (ANOVA) was used for three or more variables. (d) Multivariate analysis: Variables found to be statistically significant in the univariate analysis were included in a multiple linear regression model to analyze the factors influencing ACP knowledge, attitudes, and practice intentions separately. Categorical independent variables were treated as dummy variables, and the reference group was clearly specified in the variable coding table. Before conducting multiple linear regression analysis, the model assumptions are tested. A scatter plot is used to assess whether a linear relationship exists between the independent and dependent variables; residual histograms and Q-Q plots are used to assess whether the residuals are approximately normally distributed; residual plots are used to assess homoscedasticity; and multi-collinearity was assessed using variance inflation factors (VIFs). If no obvious violations of the regression assumptions are found, the multiple linear regression analysis proceeds. Regression results are reported using unstandardized regression coefficients, 95% confidence intervals, standardized regression coefficients, *t*-values, and *p*-values.

Given the non-probabilistic nature of this sampling strategy, statistical tests are used as exploratory tools to analyze patterns and associations within the study sample. These tests are not intended to support rigorous inferences at the population level. Therefore, *p*-values should be interpreted in conjunction with descriptive statistics, effect estimates, 95% confidence intervals, and clinical relevance.

### 2.5 Ethics

This study was conducted in accordance with the Declaration of Helsinki and was approved by the Ethics Committee of Anhui Provincial Hospital, the First Affiliated Hospital of the University of Science and Technology of China (Approval No. 2025-ky076). Before completing the online questionnaire, all participants were informed of the study purpose, anonymity, confidentiality, voluntary participation, and their right to withdraw. Informed consent was obtained electronically from all participants.

## 3 Results

### 3.1 General characteristics

A total of 720 questionnaires were submitted through the online survey platform. In accordance with predefined quality control standards, 34 questionnaires were excluded because they were completed in less than 180 s or contained repetitive responses. Ultimately, 686 valid questionnaires were included, resulting in a valid questionnaire rate of 95.28%. The final sample included 686 emergency department healthcare providers from 54 hospitals, including 160 physicians and 526 nurses. Among them, 203 were male and 483 were female. The mean age was (32.7 ± 7.19) years, with 58% of participants under 30 years, 24.5% aged 31–40, and 15.5% over 40 years. Refer to Table 1 for specifics.

**Table 1:** General information of emergency department medical staff (*n* = 686).

Item	*n* (%)
Gender	
Male	203 (29.6)
Female	483 (70.4)
Profession	
Doctor	160 (23.3)
Nurse	526 (76.7)
Hospital level	
Secondary hospital	348 (50.7)
Tertiary hospital	338 (49.3)
Professional title	
Junior	146 (21.3)
Intermediate	179 (26.1)
Associate senior or above	361 (52.6)
Education level	
Associate degree or below	142 (20.7)
Bachelor’s degree	319 (46.5)
Master’s degree or above	225 (32.8)
Marital status	
Unmarried	179 (26.1)
Married	464 (67.6)
Divorced	38 (5.5)
Widowed	5 (0.7)
Having children	
No	426 (62.1)
Yes	260 (37.9)
Age (years)	
≤30	398 (58.0)
31–40	182 (26.5)
>40	106 (15.5)
Years of emergency work (years)	
≤10	345 (50.3)
11–20	216 (31.5)
>20	125 (18.2)
Palliative/hospice care education	
None	194 (28.3)
Learned during schooling	292 (42.6)
Trained after employment	200 (29.2)
Heard of ACP	
Never heard of it	287 (41.8)
Know a little	230 (33.5)
Understand it	169 (24.6)
Experience with death	
None	196 (28.6)
1-2 times/month	301 (43.9)
3–5 times/month	100 (14.6)
>5 times/month	89 (13.0)

### 3.2 Scores of emergency department staff on ACP knowledge, attitudes, and practice intentions

The theoretical possible score range was 0–15 for the knowledge dimension, 24–120 for the attitude dimension, and 9–45 for the practice intention dimension, with a total theoretical possible score range of 33–180. Among the 686 emergency department healthcare providers, the mean total ACP KAP score was 102.72 ± 32.87. The mean scores for ACP knowledge, attitudes, and practice intentions were 7.25 ± 5.37, 73.91 ± 25.24, and 21.56 ± 10.31, respectively. These results suggest that participants generally recognized the value of ACP, but their ACP-related knowledge and practice intentions still had room for improvement. Item-level analysis showed that only 1.6% of participants answered all knowledge items correctly, whereas 6.5% answered all knowledge items incorrectly. In addition, 48.3% of participants achieved an accuracy rate of 60% or above. Participants had relatively lower accuracy for items concerning the definition of ACP, the definition of end-of-life, and the legal responsibilities of surrogate decision-makers. By contrast, items related to ACP communication processes and implementation procedures had accuracy rates above 50%. In the attitude and practice intention dimensions, 41.5% of participants regarded the emergency department as a major setting for ACP implementation, and 33.2% reported readiness to engage in ACP-related clinical tasks requiring teamwork and standardized procedures. Table 2 displays the scores for number of people who answered correctly and their corresponding accuracy rates.

**Table 2:** Correctness rate of ACP knowledge among emergency department healthcare professionals (*n* = 686).

Item	Accurate count of individuals (*n*)	Correct (%)
Top 5 items with highest correct rate (top 1/3)		
Item 12. ACP communication process	421	61.4
Item 9. Key points of ACP implementation	407	59.3
Item 15. Primary goal of implementing ACP	406	59.2
Item 11. Content of ACP discussions	399	58.2
Item 10. Timing of ACP initiation	389	56.7
Bottom 5 items with lowest correct rate (bottom 1/3)		
Item 6. Understanding the process of promoting ACP	294	42.9
Item 2. Definition of end-of-life	279	40.7
Item 5. Legally designated surrogate for advance care planning	262	38.2
Item 1. Definition of ACP	258	37.6
Item 4. Definition of advance care planning surrogate	232	33.8

### 3.3 Correlation analysis of ACP knowledge, attitudes, and practice intentions among emergency department staff

The correlation study of ACP knowledge, attitudes, and practice intentions demonstrated significant positive correlations among these three variables. The correlation coefficient between ACP knowledge and ACP attitudes was 0.548 (*p* < 0.01), between ACP knowledge and ACP practice intentions was 0.180 (*p* < 0.01), and between ACP attitude and ACP practice intentions was 0.268 (*p* < 0.01), indicating a significant positive correlation.

### 3.4 Comparison of ACP knowledge, attitudes, and practice intentions scores among emergency department personnel with varied characteristics

Exploratory univariate analyses were conducted to describe differences in ACP knowledge, attitudes, and practice intention scores across participant characteristics within the study sample. The detailed descriptive statistics and test results are presented in Table 3. Variables with *p* < 0.05 in the exploratory univariate analyses were considered for inclusion in the multivariable regression models.

**Table 3:** Univariate analysis of ACP knowledge, attitudes, and practice intentions scores among emergency department healthcare workers (*n* = 686).

Variable	Category	*n*	Knowledge	Attitudes	Practice intentions	Total score
Gender	Male	203	6.60 ± 5.50	73.90 ± 25.05	21.90 ± 10.65	102.40 ± 32.86
	Female	483	7.52 ± 5.29	73.92 ± 25.35	21.41 ± 10.17	102.85 ± 32.90
*t*			−2.06	−0.01	0.57	0.16
*p*			0.04	0.99	0.57	0.87
Age (years)	≤30	398	5.54 ± 5.00	56.53 ± 19.03	19.00 ± 9.36	81.07 ± 24.03
	31–40	182	8.02 ± 5.33	85.63 ± 19.69	23.16 ± 10.12	116.81 ± 27.14
	>40	106	11.08 ± 4.15	98.30 ± 18.88	25.56 ± 12.02	134.94 ± 26.87
*F*			44.07	244.43	20.23	216.82
*p*			0.01	<0.01	<0.01	<0.01
Marital status	Unmarried	179	7.26 ± 5.56	73.35 ± 25.14	22.34 ± 10.71	104.19 ± 31.98
	Married	464	7.20 ± 5.32	74.59 ± 25.33	21.25 ± 10.21	101.80 ± 33.08
	Divorced/widowed	43	7.72 ± 5.14	77.21 ± 26.28	21.63 ± 9.75	106.56 ± 34.42
*F*			0.18	0.55	0.72	0.65
*p*			0.83	0.58	0.49	0.52
Having children	No	426	6.93 ± 5.44	72.42 ± 25.59	20.86 ± 9.81	100.21 ± 32.97
	Yes	260	7.77 ± 5.21	76.36 ± 24.52	22.70 ± 10.99	106.83 ± 32.35
*t*			−1.98	−1.99	−2.27	−2.57
*p*			0.04	0.04	0.02	0.01
Hospital level	Secondary hospital	348	6.87 ± 5.28	71.46 ± 23.55	20.34 ± 9.60	98.68 ± 30.11
	Tertiary hospital	338	7.64 ± 5.43	76.44 ± 26.68	22.80 ± 10.86	106.89 ± 35.04
*t*			−1.9	−2.59	−3.14	−3.29
*p*			0.06	0.01	<0.01	<0.01
Years of emergency work (years)	≤10	345	6.80 ± 5.25	70.15 ± 23.42	21.06 ± 10.15	98.01 ± 30.57
	11–20	216	7.02 ± 5.50	75.19 ± 26.93	20.94 ± 9.98	103.16 ± 34.14
	>20	125	8.89 ± 5.19	82.10 ± 25.13	23.98 ± 11.00	114.97 ± 33.77
*F*			7.37	11.00	4.29	12.66
*p*			<0.01	<0.01	0.01	<0.01
Profession	Doctor	160	7.53 ± 4.99	69.10 ± 23.65	21.89 ± 10.54	98.15 ± 30.21
	Nurse	526	7.17 ± 5.48	75.38 ± 25.55	21.45 ± 10.24	104 ± 33.55
*t*			0.74	−2.77	0.47	1.85
*p*			0.46	0.01	0.64	0.06
Professional title	Junior	146	6.29 ± 4.88	67.04 ± 23.73	20.25 ± 10.06	93.58 ± 29.59
	Intermediate	179	6.56 ± 5.09	74.9 ± 28.54	21.38 ± 10.33	102.84 ± 37.45
	Associate senior or above	361	7.98 ± 5.59	76.2 ± 23.62	22.17 ± 10.37	106.36 ± 31.02
*F*			7.29	7.16	1.84	8.01
*p*			<0.01	<0.01	0.16	<0.01
Education level	Associate degree or below	142	5.58 ± 5.19	69.02 ± 25.80	20.52 ± 10.35	95.13 ± 33.38
	Bachelor’s degree	319	7.65 ± 5.35	74.45 ± 24.03	20.78 ± 9.21	102.89 ± 30.51
	Master’s degree or above	225	7.73 ± 5.31	76.24 ± 26.24	23.30 ± 11.51	107.28 ± 34.97
*F*			8.85	3.72	4.90	6.04
*p*			<0.01	0.03	0.01	<0.01
Palliative/hospice care education	None	194	6.03 ± 4.82	70.31 ± 24.19	21.59 ± 9.22	97.92 ± 29.79
	Learned during schooling	292	7.60 ± 5.58	73.25 ± 26.47	20.23 ± 10.41	101.09 ± 34.55
	Trained after employment	200	7.93 ± 5.38	78.38 ± 23.83	23.46 ± 10.89	109.76 ± 32.19
*F*			7.40	5.27	5.92	7.14
*p*			<0.01	0.01	<0.01	<0.01
Heard of ACP	Never heard of it	287	6.28 ± 5.52	69.23 ± 27.38	20.03 ± 9.38	95.54 ± 34.10
	Know a little	230	7.64 ± 5.11	75.07 ± 22.58	21.67 ± 9.17	104.38 ± 29.47
	Understand it	169	8.38 ± 5.18	80.29 ± 23.39	23.98 ± 12.60	112.65 ± 32.87
*F*			9.30	10.87	7.96	15.47
*p*			<0.01	<0.01	<0.01	<0.01
Experience with death	None	196	6.91 ± 5.06	70.61 ± 24.24	21.24 ± 10.79	98.77 ± 31.41
	1-2 times/month	301	6.78 ± 5.37	72.07 ± 24.48	21.77 ± 10.22	100.62 ± 31.87
	3–5 times/month	100	7.80 ± 5.07	82.68 ± 24.91	22.06 ± 10.46	112.54 ± 32.74
	>5 times/month	89	8.98 ± 6.00	77.57 ± 27.94	20.94 ± 9.42	107.49 ± 37.02
*F*			4.53	6.45	0.29	5.05
*p*			<0.01	<0.01	0.83	<0.01

Independent-samples *t*-tests were used for comparisons between two groups, and one-way ANOVA was used for comparisons among three or more groups. Values are presented as mean ± SD. *p* < 0.05 was considered statistically significant. The theoretical possible score ranges are 0–15 for ACP knowledge, 24–120 for ACP attitudes, 9–45 for ACP practice intentions, and 33–180 for the total KAP score. Higher scores indicate higher knowledge, more positive attitudes, and stronger practice intentions.

### 3.5 Multivariate linear regression analysis of factors influencing knowledge, attitudes, and practice intentions regarding ACP among emergency department medical staff

A multivariate linear regression analysis was performed utilizing the raw scores from the ACP knowledge, attitude, and behavior questionnaire as dependent variables, whereas statistically significant characteristics identified in the univariate study were utilized as independent variables. Quantitative data were recorded as raw values, while other independent factors were encoded as illustrated in Table 4.

**Table 4:** Assignment of independent variables.

Variable	Coding
Gender	Male = 0, female = 1
Age	Grouped as ≤30, 31–40, >40; dummy variables with ≤30 as reference
Years of emergency work	Grouped as ≤10, 11–20, >20; dummy variables with ≤10 as reference
Professional title	Grouped as junior, intermediate, associate senior or above; dummy variables with junior as reference
Education level	Grouped as associate degree or below, bachelor’s degree, master’s degree or above; dummy variables with associate degree or below as reference
Hospital level	Tertiary hospital = 0, secondary hospital = 1
Profession	Doctor = 0, nurse = 1
Children	No = 0, yes = 1
Palliative/hospice care education experience	Grouped as none, learned during schooling, trained after employment; dummy variables with none as reference
Awareness of ACP	Grouped as never heard, know a little, understand it; dummy variables with never heard as reference
Experience with death	Grouped as none, 1-2 times/month, 3–5 times/month, >5 times/month; dummy variables with none as reference

The dependent variables were raw scores for ACP knowledge, attitudes, and practice intentions. Their theoretical possible score ranges were 0–15, 24–120, and 9–45, respectively; the theoretical total KAP score range was 33–180.

Multiple linear regression analysis showed that several factors were associated with ACP knowledge scores. Female emergency department healthcare providers had higher knowledge scores than male providers (*B* = 1.030, 95% CI: 0.231 to 1.828, *β* = 0.088, *t* = 2.526, *p* = 0.012). Compared with participants aged ≤30 years, those aged 31–40 years (*B* = 2.110, 95% CI: 1.308 to 2.951, *β* = 0.195, *t* = 5.012, *p* < 0.001) and >40 years (*B* = 4.124, 95% CI: 2.857 to 5.378, *β* = 0.247, *t* = 6.210, *p* < 0.001) had higher ACP knowledge scores. Participants with a bachelor’s degree (*B* = 1.666, 95% CI: 0.657 to 2.637, *β* = 0.155, *t* = 3.294, *p* = 0.001) or a master’s degree or above (*B* = 1.616, 95% CI: 0.562 to 2.641, *β* = 0.142, *t* = 3.041, *p* = 0.002) also had higher knowledge scores than those with an associate degree or below. Compared with participants with ≤10 years of emergency work experience, those with >20 years of experience had higher ACP knowledge scores (*B* = 1.214, 95% CI: 0.246 to 2.276, *β* = 0.087, *t* = 2.329, *p* = 0.020). Palliative/hospice care education was also associated with higher ACP knowledge, including education received during schooling (*B* = 1.284, 95% CI: 0.403 to 2.213, *β* = 0.118, *t* = 2.773, *p* = 0.006) and training after employment (*B* = 1.162, 95% CI: 0.169 to 2.151, *β* = 0.098, *t* = 2.296, *p* = 0.022). In addition, participants who knew a little about ACP (*B* = 1.309, 95% CI: 0.476 to 2.221, *β* = 0.115, *t* = 2.925, *p* = 0.004) or understood ACP (*B* = 1.066, 95% CI: 0.128 to 2.091, *β* = 0.086, *t* = 2.108, *p* = 0.035) had higher knowledge scores than those who had never heard of ACP. Participants who managed more than five death-related cases per month also had higher knowledge scores than those without such experience (*B* = 1.497, 95% CI: 0.256 to 2.738, *β* = 0.094, *t* = 2.359, *p* = 0.019). The knowledge model showed acceptable explanatory power, with *R*^2^ = 0.243 and adjusted *R*^2^ = 0.205, indicating that the model explained 20.5% of the variance in ACP knowledge scores. The overall model was statistically significant (*F* = 6.355, *p* < 0.001).

For ACP attitudes, age, years of emergency work, profession, awareness of ACP, and experience with death were associated with attitude scores. Compared with participants aged ≤30 years, those aged 31–40 years (*B* = 28.520, 95% CI: 25.481 to 31.544, *β* = 0.560, *t* = 18.448, *p* < 0.001) and >40 years (*B* = 41.398, 95% CI: 36.593 to 46.156, *β* = 0.527, *t* = 16.978, *p* < 0.001) had more positive attitudes toward ACP. Participants with >20 years of emergency work experience also had higher attitude scores than those with ≤10 years of experience (*B* = 4.567, 95% CI: 0.805 to 8.292, *β* = 0.070, *t* = 2.386, *p* = 0.017). Nurses had higher ACP attitude scores than physicians (*B* = 12.954, 95% CI: 9.586 to 16.290, *β* = 0.217, *t* = 7.569, *p* < 0.001). Participants who understood ACP had higher attitude scores than those who had never heard of ACP (*B* = 6.138, 95% CI: 2.574 to 9.814, *β* = 0.105, *t* = 3.307, *p* = 0.001). Compared with participants without experience of managing death-related cases, those who managed death-related cases 3–5 times per month (*B* = 8.935, 95% CI: 4.588 to 13.285, *β* = 0.125, *t* = 4.026, *p* < 0.001) and more than five times per month (*B* = 5.550, 95% CI: 0.994 to 10.116, *β* = 0.074, *t* = 2.382, *p* = 0.018) had higher attitude scores. The attitude model had *R*^2^ = 0.539 and adjusted *R*^2^ = 0.516, indicating that it explained 51.6% of the variance in ACP attitude scores. The overall model was statistically significant (*F* = 23.125, *p* < 0.001).

For ACP practice intentions, age, hospital level, and awareness of ACP were associated with practice intention scores. Compared with participants aged ≤30 years, those aged 31–40 years (*B* = 3.689, 95% CI: 2.076 to 5.251, *β* = 0.177, *t* = 4.342, *p* < 0.001) and >40 years (*B* = 4.563, 95% CI: 2.032 to 7.049, *β* = 0.142, *t* = 3.405, *p* = 0.001) had stronger ACP practice intentions. Compared with participants from tertiary hospitals, those from secondary hospitals had lower practice intention scores (*B* = −2.162, 95% CI: −3.646 to −0.705, *β* = −0.105, *t* = −2.864, *p* = 0.004). Participants who understood ACP had higher practice intention scores than those who had never heard of ACP (*B* = 2.469, 95% CI: 0.592 to 4.532, *β* = 0.103, *t* = 2.420, *p* = 0.016). The practice intention model had *R*^2^ = 0.165 and adjusted *R*^2^ = 0.123, indicating that it explained 12.3% of the variance in ACP practice intention scores. The overall model was statistically significant (*F* = 3.906, *p* < 0.001). The results of the multiple linear regression analyses are shown in Table 5.

**Table 5:** Multivariate linear regression analysis of ACP knowledge, attitudes, and practice intentions among emergency department healthcare providers.

Variable	*B*	SE	β	*t*	*p*	95% CI
Model 1: knowledge dimension (constant)	0.311	1.133	—	0.275	0.784	—
Gender (ref: male)						
Female	1.030	0.408	0.088	2.526	0.012	0.231–1.828
Age (ref: ≤ 30 years)						
31–40 years	2.110	0.421	0.195	5.012	<0.001	1.308–2.951
>40 years	4.124	0.664	0.247	6.210	<0.001	2.857–5.378
Years of emergency work (ref: ≤ 10 years)						
>20 years	1.214	0.521	0.087	2.329	0.020	0.246–2.276
Education level (ref: associate degree or below)						
Bachelor’s degree	1.666	0.506	0.155	3.294	0.001	0.657–2.637
Master’s degree or above	1.616	0.531	0.142	3.041	0.002	0.562–2.641
Palliative/hospice care education (ref: none)						
Learned during schooling	1.284	0.463	0.118	2.773	0.006	0.403–2.213
Trained after employment	1.162	0.506	0.098	2.296	0.022	0.169–2.151
Awareness of ACP (ref: never heard)						
Know a little	1.309	0.448	0.115	2.925	0.004	0.476–2.221
Understand it	1.066	0.506	0.086	2.108	0.035	0.128–2.091
Experience with death (ref: none)						
>5 times/month	1.497	0.635	0.094	2.359	0.019	0.256–2.738
Model 2: attitude dimension (constant)	31.402	4.160	—	7.549	<0.001	—
Age (ref: ≤30 years)						
31–40 years	28.520	1.546	0.560	18.448	<0.001	25.481–31.544
>40 years	41.398	2.438	0.527	16.978	<0.001	36.593–46.156
Years of emergency work (ref: ≤10 years)						
>20 years	4.567	1.914	0.070	2.386	0.017	0.805–8.292
Profession (ref: doctor)						
Nurse	12.954	1.712	0.217	7.569	<0.001	9.586–16.290
Awareness of ACP (ref: never heard)						
Understand it	6.138	1.856	0.105	3.307	0.001	2.574–9.814
Experience with death (ref: none)						
3–5 times/month	8.935	2.219	0.125	4.026	<0.001	4.588–13.285
>5 times/month	5.550	2.330	0.074	2.382	0.018	0.994–10.116
Model 3: practice intentions dimension (constant)	15.618	2.286	—	6.832	<0.001	—
Age (ref: ≤30 years)						
31–40 years	3.689	0.850	0.177	4.342	<0.001	2.076–5.251
>40 years	4.563	1.340	0.142	3.405	0.001	2.032–7.049
Hospital level (ref: tertiary)						
Secondary	−2.162	0.755	−0.105	−2.864	0.004	−3.646 to −0.705
Awareness of ACP (ref: never heard)						
Understand it	2.469	1.020	0.103	2.420	0.016	0.592–4.532

*B*, unstandardized regression coefficient; SE, standard error; *β*, standardized regression coefficient; CI, confidence interval; The 95% CI refers to the unstandardized regression coefficient *B*. Only statistically significant predictors retained in the final regression models are presented. *p* < 0.05 was considered statistically significant.

## 4 Discussion

### 4.1 Emergency department healthcare providers showed insufficient ACP knowledge, particularly regarding core concepts and legal-ethical issues

The findings showed that emergency department healthcare providers had a mean ACP knowledge score of 7.25 ± 5.37 out of a possible 15 points, suggesting that their ACP-related knowledge requires further improvement. This corresponds with earlier domestic research indicating significant deficiencies in ACP knowledge (12, 18, 19). Comprehensive analysis indicated that emergency department personnel exhibited a basic comprehension of “what actions are necessary” and “how to broadly execute” ACP. Nevertheless, there was a notable deficiency in comprehension about the fundamental principles that underpin the legal and ethical framework of ACP practice This knowledge framework, defined by an understanding of “what” but not “why,” fundamentally contributes to their “challenges in effectively engaging in ACP-related discussions.” Current ACP training frequently prioritizes procedural steps and communication tactics while insufficiently covering essential ideas such as ACP’s fundamental definitions, philosophical foundations, legal validity, and the ethical limits of “end of life” (2). The distinctive operational tempo of emergency departments is another critical element. Emergency rooms, in contrast to general wards or specialist care settings, present elevated work pressure and a more rapid pace, necessitating doctors to navigate more intricate and dynamic clinical situations while making vital judgments under constrained timelines (20). This setting hinders methodical conceptual learning and profound thought. Consequently, structured training modules must be established that concentrate on prevalent emergency scenarios and reaffirm fundamental ideas, including key definitions, legal considerations, and ethical principles of ACP. Screening trigger mechanisms and standardized communication protocols suited to the emergency department’s tempo must be implemented. Simultaneously, interdisciplinary support teams that include ethics and legal consultants, along with explicit institutional safeguards, must be established to effectively overcome the existing knowledge gap.

### 4.2 Emergency department healthcare providers held relatively positive attitudes toward ACP but remained concerned about its implementation

The mean ACP attitude score was 73.91 ± 25.24 out of a possible 120 points, indicating that emergency department healthcare providers generally recognized the value of ACP. This is closely related to the recognition of ACP’s clinical benefits for terminally ill patients, the value of patient autonomy, and its role in alleviating the burden on families and society (21–23). This recognition may have contributed to their conceptual acceptance of ACP and their perceived need for structured training. They generally held the view that “early ACP education” and “enhancing ACP training for healthcare providers” might facilitate ACP adoption, while also recognizing their responsibilities in communication. The present findings suggest that lower scores on items related to traditional filial piety, death-related taboos, and the influence of family culture on patient autonomy may reflect the continuing influence of sociocultural factors on ACP communication. In accordance with previous research (24–26), emergency department healthcare providers articulated considerable apprehension and ambiguity when faced with particular ethical considerations, cultural conflicts, and legal challenges related to the implementation of ACP. The contradiction lies in the fact that while healthcare providers generally support ACP, the high uncertainty and emotionally charged decision-making environment unique to emergency settings often prevents patients from expressing their wishes. In emergency situations, family members may experience intense emotional distress and may have difficulty accepting discussions about withholding or withdrawing life-sustaining treatment and end-of-life preferences (27). Healthcare providers may also worry about being misunderstood as providing “negative treatment,” which could increase perceived risk in clinician–patient or clinician-family communication. The disparity between theoretical consensus and practical reluctance obstructs the effective execution of ACP in emergency rooms and may lead to insufficient regard for the treatment preferences of terminally ill patients. Concurrently, it exacerbates the ethical issues and decision-making demands encountered by healthcare providers (28). Consequently, it is essential to create explicit policy and legal frameworks that define the implementation requirements for ACP in emergency contexts and clarify the scope of healthcare professionals’ responsibilities to assure legal validity. A training system designed for emergency clinical contexts must be established, emphasizing the development of practical skills including challenging communication strategies, ethical dilemma resolution, and cultural collaboration in emergency situations. Enhance public education on ACP to progressively alter conventional beliefs and cultivate a societal environment that comprehends and honors the treatment preferences of terminally ill patients. Simultaneously, healthcare organizations must facilitate ACP implementation by enhancing emergency service processes, judiciously allocating medical resources, and providing dedicated time and place for ACP dialogues. This will translate healthcare professionals’ value recognition into safe and effective clinical decision-making practice intentions.

### 4.3 ACP practice intentions among emergency department healthcare providers remained limited

The mean ACP practice intention score was 21.56 ± 10.31 out of a possible 45 points, suggesting that participants’ willingness to engage in ACP-related practice was not yet strong. This corresponds with their inadequate understanding and ambiguous attitudes on ACP (29). These findings suggest that limited ACP practice intentions may not simply reflect rejection of ACP as a concept, but may also be related to the difficulty of integrating ACP-related work into a high-pressure, fast-paced, life-saving-oriented emergency setting. Healthcare providers may be more willing to engage in low-risk or individualized ACP-related communication, but less prepared to participate in ACP-related tasks that require institutional support, interdisciplinary collaboration, documentation, or clear responsibility allocation. Therefore, practical support systems may be needed, including electronic documentation tools, emergency-specific ACP guidance, clear role allocation, and multidisciplinary collaboration mechanisms. Simultaneously, improve multidisciplinary collaboration mechanisms to alleviate liability concerns and individual pressures among emergency department medical staff. Mitigate knowledge deficiencies and resolve attitudinal discrepancies among emergency department personnel with specialized training initiatives. Mitigate situational opposition to ACP among emergency department staff to facilitate the effective execution of ACP in emergency contexts.

### 4.4 Multiple factors were associated with ACP knowledge, attitudes, and practice intentions among emergency department healthcare providers

According to the KAP model, knowledge is essential for the development of attitudes and the alteration of practice intentions (30). This study reveals that while emergency department healthcare personnel possess generally favorable attitudes toward ACP, their knowledge and desire to implement it are inadequate, indicating that their acknowledgment of ACP’s significance has not been effectively converted into clinical practice. The factors contributing to this disparity may be associated with the distinctive environment of the emergency department. Due to the rapid work tempo, abrupt alterations in patient status, and constrained decision-making time, healthcare providers frequently emphasize life support and acute symptom management, hindering comprehensive communication about care objectives (31). Moreover, family members in emergency situations frequently exhibit significant anxiety and demonstrate limited acceptance of end-of-life decisions, including the restriction or cessation of ineffective treatments, hence exacerbating healthcare providers’ reluctance to participate in advance care planning discussions (32). Therefore, the practice of ACP in emergency departments requires clear identification criteria, standardized communication tools, multidisciplinary support, and explicit legal and ethical safeguards.

### 4.5 The influence of gender, age, and years of emergency department experience on knowledge, attitudes, and practice intentions regarding ACP

This study reveals that female healthcare providers in emergency departments possess greater understanding of ACP than their male counterparts. In clinical settings, women often shoulder more responsibilities in communication, patient education, patient care, and documentation, and are more frequently exposed to ACP processes and training content. A study conducted in Saudi Arabia about advance directives among healthcare professionals revealed that female physicians and nurses attained superior knowledge scores (33). Moreover, research demonstrates that female physicians are more inclined to commence ACP talks and obtain formal ACP training (34). Nevertheless, alternative research indicates that the correlation between gender and ACP knowledge is inconsistent, presumably affected by confounding variables such as professional composition, work environments, and training opportunities (35). This is consistent with the findings of Huang (36). As age advances and years of emergency department experience mount, healthcare providers acquire enhanced clinical skill and life experience, allowing for a deeper understanding of ACP theory and its significance. This may partly explain their higher ACP knowledge and more favorable attitudes. However, certain research suggest that younger healthcare professionals, having undergone contemporary medical ethics training, exhibit more acceptance of innovative concepts such as ACP (37). While seasoned emergency department healthcare providers exhibit more involvement with ACP, younger practitioners hold much promise. Young individuals generally demonstrate robust learning capabilities and adaptability, providing significant potential for the acquisition of ACP knowledge and its practical application. Consequently, future training should deliver personalized instruction customized for various age demographics, particularly affording younger healthcare workers increased opportunity for ACP-related knowledge acquisition and practical experience to deepen their comprehension of ACP.

### 4.6 The influence of hospital level and professional role on attitudes and practice intentions regarding advance care planning

Emergency ACP practice intentions and behaviors are influenced by system and position factors. This study reveals that emergency department personnel in tertiary hospitals exhibit higher levels of ACP engagement than those in secondary hospitals. Tertiary hospitals have superior training resources, policy pilot programs, multidisciplinary teams, and institutional processes, offering the knowledge, skills, and organizational support essential for standardized ACP implementation. Moreover, increased trust among patients and families in tertiary hospitals enhances collaborative advance care planning discussions between clinicians and patients. In contrast to physicians, emergency nurses-who engage more often with patients and families in providing information, emotional support, and coordinating communication—directly perceive the benefits of ACP in mitigating conflicts and improving decision quality, frequently demonstrating more favorable attitudes toward ACP. Prior study also demonstrates that emergency nurses broadly acknowledge the significance of engaging in ACP talks within emergency environments (38). A comparison study (33) on nurses’ and physicians’ perspectives and attitudes concerning advance directives revealed that nurses exhibited considerably higher attitude scores. Consequently, future initiatives might leverage the experiences of tertiary hospitals by enhancing training and system development, elucidating processes and team roles, to facilitate the introduction and general adoption of ACP in larger healthcare environments.

### 4.7 The influence of educational attainment and awareness of ACP-related content on knowledge, attitudes, and practice intentions regarding ACP

Research indicates that emergency department medical staff with higher educational qualifications demonstrate a stronger grasp of ACP knowledge. Similar to the findings of Ding et al. (39), greater educational levels among emergency department personnel indicate more systematic foundational training, a robust theoretical framework, and improved competencies in information retrieval and integration. These traits enhance their understanding of ACP’s fundamental concepts, ethical principles, and communication frameworks, allowing them to convert guidelines and evidence into practical clinical decision support. Individuals with lower educational attainment may encounter obstacles in acquiring, comprehending, and implementing ACP information due to comparatively poorer theoretical underpinnings and restricted learning resources, thereby limiting its application in practical work. The depth of comprehension of ACP’s essence directly affects the knowledge, attitudes, and practice intentions of emergency department personnel. Relevant findings (23, 40) indicate that those with enhanced ACP cognition are more likely to establish stable value identification and practice motivation. They actively emphasize patient autonomy and personalized medical decisions in clinical communication, converting concepts into tangible actions, leading to enhancements in knowledge, attitudes, and behaviors. The ACP knowledge level of emergency department healthcare providers is affected by their fundamental education and previous understanding. Fundamental education augments the ability to comprehend and acquire knowledge regarding ACP, while existing knowledge offers guidance and impetus for urgent ACP initiatives. Consequently, training and management must concurrently tackle competency deficiencies and cognitive reinforcement. For emergency department personnel with limited educational backgrounds, prioritize clear explanations of fundamental ideas and practical operational training. Augment scenario simulations, bedside instruction, and practice with established communication protocols. Offer sustainable departmental assistance and pragmatic possibilities to enable the conversion of knowledge into action. Encourage highly educated healthcare providers to undertake leadership and mentorship positions. By means of demonstration, oversight, and the exchange of experiences, they can facilitate collective team enhancement and promote the standardized execution of ACP in the emergency department.

### 4.8 The influence of specialized training on knowledge, attitudes, and practice intentions regarding ACP

This study reveals that emergency department healthcare providers with palliative/hospice care education exhibit enhanced awareness of ACP. This training not only enriches their comprehension of the advantages (41) and significance of ACP but also significantly improves their communication abilities, hence augmenting their confidence in ACP-related dialogues (42) and facilitating more active participation in ACP. Research by Nong et al. (43) demonstrates that structured training can enhance healthcare personnel’ confidence in executing ACP communication. Nevertheless, the majority of existing ACP training programs are tailored for general wards and are not suited for the rapid, high-stress context of emergency rooms. Consequently, future training systems ought to improve situational flexibility, augment end-of-life communication competencies, address experience disparities, and implement a structured division of labor with standardized documentation templates. This method minimizes time and liability concerns, enhancing the practicality and sustainability of ACP implementation in emergency contexts.

### 4.9 The influence of special experiential factors on knowledge, attitudes, and practice intentions regarding ACP

In this study, more frequent experience with death-related cases was associated with higher ACP knowledge scores and more positive attitudes toward ACP. One possible explanation is that repeated exposure to end-of-life situations may increase healthcare providers’ awareness of treatment boundaries, patient preferences, and the potential consequences of non-beneficial treatment. This interpretation is broadly consistent with previous findings suggesting that death-related clinical experiences may prompt healthcare professionals to reflect more deeply on mortality, end-of-life communication, and the quality of care decisions (15). However, this association should be interpreted cautiously. Alternative explanations are also possible. For example, healthcare providers who are already more interested in end-of-life care or ACP may be more attentive to death-related clinical situations and more likely to report such experiences. In addition, repeated exposure to death does not necessarily increase willingness to participate in ACP. Without sufficient psychological support, frequent exposure to death and high-intensity emergency work may contribute to compassion fatigue, burnout, or emotional exhaustion, which could reduce healthcare providers’ readiness to engage in complex ACP-related communication (7, 20). Therefore, death-related experience should not be viewed as a direct cause of better ACP knowledge or more positive attitudes. Rather, it may represent a clinical context in which healthcare providers become more aware of the need for structured end-of-life communication. Future ACP training in emergency departments should help healthcare providers transform clinical experience into standardized communication practice while also providing psychological support and stress-reduction strategies. Given the cross-sectional design of this study, the observed associations should be regarded as hypothesis-generating, and future longitudinal or qualitative studies are needed to further explore how death-related clinical experiences shape ACP knowledge, attitudes, and practice intentions.

Because this study used a cross-sectional design, all explanatory interpretations of the observed associations should be understood as hypothesis-generating rather than causal conclusions.

This study does not contend that emergency departments should perform the complete ACP procedure at the resuscitation site; instead, it emphasizes the necessity for emergency department personnel to recognize ACP requirements and initiate preliminary communication. Emergency departments are more adept at “identifying patient needs, facilitating initial communication, documenting patient preferences, and referring patients to palliative care or ethics teams.” Hospital systems and lines of responsibility must be developed; legal and ethical support at the policy level should be enhanced; and future research should assess the efficacy of these initiatives.

## 5 Limitations of the study and future recommendations

This study has several limitations. First, strict probability-based random sampling was not used. Hospitals were selected using a stratified quota-based approach, and participants were recruited through convenience sampling within participating emergency departments. Therefore, selection bias and unit non-response bias cannot be excluded. Because the exact number of eligible healthcare providers who received or viewed the survey invitation could not be determined, the reported proportion should be interpreted as a valid questionnaire rate rather than a strict response rate. Second, reliable province-wide workforce data for emergency department healthcare providers were not available; therefore, the representativeness of the sample could not be formally assessed, and weighting procedures were not performed. The findings should be interpreted as exploratory evidence from the participating sample rather than as population-level estimates. Third, this study used a self-administered online questionnaire. Although the survey was closed and the platform was configured to allow only one submission from the same IP address, no independent log-file analysis was conducted. Repeated submissions, recall bias, and social desirability bias cannot be completely ruled out. Fourth, the cross-sectional design precludes causal inference. The observed associations should be interpreted as hypothesis-generating rather than causal. In addition, potential center effects were not fully addressed using multilevel modeling or cluster-robust estimation. Finally, although the adapted ACP KAP questionnaire showed good internal consistency, further validation is needed in emergency department populations.

Future studies should include larger and more diverse samples across different regions and healthcare settings, use more rigorous sampling strategies, and consider longitudinal, mixed-methods, and intervention designs. Research involving healthcare providers, patients, family caregivers, administrators, palliative care specialists, and ethics or legal professionals may provide a more comprehensive understanding of ACP implementation in emergency settings. Emergency-specific ACP training, screening triggers, communication protocols, documentation tools, referral pathways, and multidisciplinary support models should be developed and evaluated in future research.

## 6 Conclusion

Emergency department healthcare providers in China recognized the importance of ACP, but their ACP-related knowledge and practice intentions remained limited. ACP-related work in emergency departments should focus on identifying potential needs, initiating brief and appropriate communication, documenting care preferences, and facilitating referrals, rather than completing a comprehensive ACP process during acute crisis care. Emergency-specific training, standardized procedures, multidisciplinary support, and clearer legal and ethical safeguards are needed to support feasible ACP-related practice in emergency settings.

## Data Availability

The datasets generated and analyzed during the current study are not publicly available due to ethical and institutional restrictions. The participating institutions did not grant permission for public data sharing. Reasonable requests for access to the data may be directed to the corresponding author and will be considered in accordance with ethical approval and institutional requirements.
