# Supplementary material for: Knowledge, attitudes, and practices regarding advance care planning among emergency department healthcare providers in China: a multicenter cross-sectional study
Source: Front Public Health. 2026 Jun 15;14:1815766. doi: 10.3389/fpubh.2026.1815766 (PMC13310977; doi:10.3389/fpubh.2026.1815766)
Supplement: Supplementary file 1 [file Data_Sheet_1.DOCX]

**Supplementary Appendix 1**

**Questionnaire on Knowledge, Attitudes, and Practice Intentions Regarding Advance Care Planning Among Emergency Department Healthcare Providers**

**I. Instructions**

Dear healthcare colleagues,

Thank you very much for completing this questionnaire. We are the research team led by Professor Chengwen Hu from the First Affiliated Hospital of the University of Science and Technology of China (Anhui Provincial Hospital). We are conducting a study entitled "Survey on Knowledge, Attitudes, and Practice Intentions Regarding Advance Care Planning (ACP) Among Emergency Department Healthcare Providers," with the aim of providing evidence for the development of ACP-related training and intervention programs for emergency department healthcare providers.

Advance care planning (ACP) refers to the process in which patients, while conscious and capable of decision-making, express their preferences for medical treatment and nursing care that they would be willing to receive in the future when they enter an end-of-life condition, based on their personal life experiences and values, after being informed about their illness, prognosis, and possible end-of-life care measures. This process also involves communicating these wishes with healthcare professionals and/or family members or friends.

After reading the instructions, please select or complete the relevant items according to your actual situation. Your personal information and responses will be kept strictly confidential, and the data will be used only for research purposes. If you have any questions or suggestions regarding this questionnaire, please feel free to contact us.

Finally, we sincerely thank you for your participation and support. We wish you success in your work and happiness in your life.

Contact person: Wenya Bi; Tel: 18895347319

**II. Informed Consent**

I fully understand the purpose, nature, methods, my rights, and any possible risks of participating in this survey. I understand that this survey does not collect personally identifiable information, that the research data will be kept confidential, and that my privacy will be protected. I voluntarily agree to participate in this survey and agree to complete the questionnaire honestly and carefully.

① Yes ② No

**Part 1. General Information Questionnaire**

1. Your gender: ① Male ② Female

2. Your age: ______ years

3. Level of the hospital where you currently work: ① Tertiary hospital ② Secondary hospital

4. Years of work experience in the emergency department: ______ years

5. Your profession: ① Physician ② Nurse

6. Your professional title: ① Junior ② Intermediate ③ Associate senior or above

7. Your highest educational level: ① Associate degree or below ② Bachelor's degree ③ Master's degree or above

8. Your marital status: ① Unmarried ② Married ③ Divorced ④ Widowed

9. Children: ① No ② Yes, ______ child(ren)

10. Have you received education or training related to palliative medicine, palliative care, hospice care, or end-of-life care in the emergency department? ① None ② Learned during schooling ③ Trained after employment

11. Before this survey, had you heard of the concepts or content of advance care planning (ACP), advance directives (AD), or living wills? ① Yes, I understand their meanings ② Yes, but I do not fully understand their meanings ③ No, I had not heard of them

12. In your emergency department work, have you ever managed the death of patients with terminal illness or age-related frailty? ① No ② 1–2 times/month ③ 3–5 times/month ④ More than 5 times/month

**Part 2. ACP Knowledge Questionnaire for Emergency Department Healthcare Providers**

**Explanation of related concepts:**

① Advance care planning (ACP): A process in which patients, while conscious and capable of decision-making, express their preferences for future medical treatment and nursing care when they enter an end-of-life condition, based on their personal life experiences and values, after obtaining information about their illness, prognosis, and possible end-of-life care measures. This process also involves communicating these wishes with healthcare professionals and/or family members or friends.

② Advance directives (AD/ADs): Documents made by individuals while conscious and capable of decision-making that state the medical treatments they would accept or refuse, their personal values and beliefs, and/or their designated healthcare proxy if they lose decision-making capacity in the future.

③ Living will (LW): A written instruction signed by individuals when healthy or conscious, specifying which medical treatments or nursing care they would or would not want at the end stage of an incurable disease or injury or when approaching death.

Instructions: The following questions concern your understanding of ACP-related knowledge. Please select the answer that best matches your understanding.

A1. ACP is a process in which physicians communicate with patients and family members about patients’ personal preferences for future health and personal care when patients are conscious and have autonomous decision-making capacity.

① True ② False ③ Do not know

A2. According to the public welfare website “Choice and Dignity,” end of life refers to a condition caused by illness or injury in which, according to reasonable medical judgment, death is generally expected to occur within six months regardless of what medical measures are used.

① True ② False ③ Do not know

A3. The validity of a living will means that it takes effect only when the person who made the will is at the end of life, in an irreversible coma, or in a persistent vegetative state.

① True ② False ③ Do not know

A4. An advance healthcare proxy refers to a person entrusted by a patient while the patient is conscious to make medical decisions on the patient’s behalf when the patient loses decision-making capacity.

① True ② False ③ Do not know

A5. An advance healthcare proxy may be a patient’s immediate family member or a person designated in accordance with the Civil Code.

① True ② False ③ Do not know

A6. The process of promoting ACP can be understood as another form of implementing euthanasia.

① True ② False ③ Do not know

A7. Patients with terminal cancer are one of the target populations for ACP implementation.

① True ② False ③ Do not know

A8. Healthcare professionals are the main implementers who encourage patients to participate in ACP and guide the ACP process.

① True ② False ③ Do not know

A9. The key focus of ACP implementation is the discussion process, which promotes effective communication among patients, families, and healthcare providers and helps determine patients’ goals for end-of-life care.

① True ② False ③ Do not know

A10. ACP should preferably be initiated as early as possible, when patients still have cognitive and decision-making capacity, so that they can make decisions more consistent with their own preferences.

① True ② False ③ Do not know

A11. Even after the content of ACP discussions has been established, it can still be modified or cancelled according to the patient’s wishes.

① True ② False ③ Do not know

A12. The ACP communication process mainly guides patients to think about their life values, beliefs, medical goals, and preferences for different treatments.

① True ② False ③ Do not know

A13. When implementing ACP, assisting patients in signing advance directives (ADs) or living wills (LWs) is not the primary goal.

① True ② False ③ Do not know

A14. For this item, please select the third option.

① True ② False ③ Do not know

A15. The goal of ACP implementation is to guide a shared decision-making process among patients, healthcare professionals, and healthcare proxies based on the patient’s wishes.

① True ② False ③ Do not know

A16. The goals of ACP implementation also include improving patient outcomes and quality of life by reducing both overtreatment and undertreatment.

① True ② False ③ Do not know

**Part 3. ACP Attitude Questionnaire for Emergency Department Healthcare Providers**

| **Item** | **Strongly agree** | **Agree** | **Uncertain** | **Disagree** | **Strongly disagree** |
| --- | --- | --- | --- | --- | --- |
| B1. Emergency department healthcare providers are the main workforce for implementing ACP in emergency departments. |  |  |  |  |  |
| B2. Emergency department healthcare providers’ views on death may affect whether they are willing to engage in ACP-related work in emergency departments. |  |  |  |  |  |
| B3. Emergency department healthcare providers should actively guide patients with terminal illness to express their treatment preferences. |  |  |  |  |  |
| B4. Emergency department healthcare providers should actively communicate and discuss patients’ treatment preferences with family members. |  |  |  |  |  |
| B5. If inconsistency is found between the wishes of patients and family members, emergency department healthcare providers should respect patients’ wishes and actively promote communication between them to reach consensus as much as possible. |  |  |  |  |  |
| B6. It is not recommended that patients with terminal illness receive treatments that excessively increase suffering without changing the final outcome, such as CPR, endotracheal intubation, or tracheotomy. |  |  |  |  |  |
| B7. Under any circumstances, patients have the greatest right to know their own treatment plans. |  |  |  |  |  |
| B8. Most surrogate decision-makers and family members have insufficient knowledge of ACP. |  |  |  |  |  |
| B9. There are seven days in a week. |  |  |  |  |  |
| B10. Due to heavy workload, it is difficult for emergency department healthcare providers to repeatedly and proactively communicate about ACP with patients and surrogate decision-makers. |  |  |  |  |  |
| B11. Insufficient knowledge of ACP among emergency department healthcare providers may hinder the clinical implementation of ACP in emergency departments. |  |  |  |  |  |
| B12. Emergency department healthcare providers lack knowledge and skills regarding how to initiate ACP conversations. |  |  |  |  |  |
| B13. Patients with terminal illness lack understanding of their own condition, believe that new treatments may prolong their lives, and are unwilling to discuss ACP. |  |  |  |  |  |
| B14. Because there is currently no legal support in China, emergency department healthcare providers are concerned that implementing ACP in emergency departments may lead to disputes. |  |  |  |  |  |
| B15. China’s traditional culture of avoiding discussion of death may hinder clinical ACP communication. |  |  |  |  |  |
| B16. China’s traditional family culture may affect patients’ decision-making autonomy; family members may conceal the patient’s condition with the intention of protecting the patient and thereby make decisions on the patient’s behalf. |  |  |  |  |  |
| B17. Under the influence of Chinese filial piety ethics, close relatives of patients may be unwilling to give up ineffective treatment due to ethical pressure. |  |  |  |  |  |
| B18. Public confusion of the concepts of “living will,” “hospice care,” and “euthanasia” is an important reason why ACP promotion is difficult. |  |  |  |  |  |
| B19. The implementation of ACP in emergency departments helps maintain patient autonomy and protect the dignity of end-of-life patients. |  |  |  |  |  |
| B20. ACP helps reduce the ethical and moral pressure on family members. |  |  |  |  |  |
| B21. The implementation of ACP in emergency departments can avoid ineffective resuscitation and reduce the economic burden on families and society. |  |  |  |  |  |
| B22. ACP can avoid disagreements between healthcare providers and patients’ families regarding treatment opinions and, to some extent, reduce the occurrence of medical disputes. |  |  |  |  |  |
| B23. Early ACP education for patients with terminal illness and their families can promote the clinical implementation of ACP. |  |  |  |  |  |
| B24. For this item, please select “Agree.” |  |  |  |  |  |
| B25. ACP knowledge and skills training for emergency department healthcare providers can promote the implementation of ACP in emergency departments. |  |  |  |  |  |
| B26. Supportive policies and regulations issued by the government can promote the implementation of ACP. |  |  |  |  |  |

**Part 4. ACP Practice Intention Questionnaire for Emergency Department Healthcare Providers**

| **Item** | **Strongly willing** | **Willing** | **Uncertain** | **Unwilling** | **Strongly unwilling** |
| --- | --- | --- | --- | --- | --- |
| C1. I am willing to place ACP brochures or books in the emergency department or play ACP-related videos. |  |  |  |  |  |
| C2. I am willing to introduce ACP-related knowledge to patients at an appropriate time. |  |  |  |  |  |
| C3. I am willing to introduce ACP-related knowledge to family members at an appropriate time. |  |  |  |  |  |
| C4. I am willing to learn and discuss ACP-related knowledge together with my colleagues. |  |  |  |  |  |
| C5. I am willing to actively carry out ACP-related clinical work in the emergency department together with my colleagues. |  |  |  |  |  |
| C6. I am willing to log in to the “Choice and Dignity” website and sign a living will for myself. |  |  |  |  |  |
| C7. I am willing to discuss my own preferences for end-of-life medical treatment and nursing care with my family. |  |  |  |  |  |
| C8. I am willing to promote ACP and encourage my relatives and friends to learn about ACP. |  |  |  |  |  |
| C9. I am willing to participate in ACP-related social activities to improve public understanding of ACP. |  |  |  |  |  |

Thank you again for your support!
